# Best practices for clinical trials of deep brain stimulation for neuropsychiatric indications

**DOI:** 10.3389/fnhum.2025.1572972

**Published:** 2025-04-16

**Authors:** Alexandra G. Tremblay-McGaw, Elissa J. Hamlat, Natalie C. Becker, Daniela A. Astudillo Maya, Andrew D. Krystal, Kristin K. Sellers

**Affiliations:** ^1^Department of Psychiatry and Behavioral Sciences, University of California, San Francisco, San Francisco, CA, United States; ^2^Weill Institute for Neurosciences, University of California, San Francisco, San Francisco, CA, United States; ^3^Department of Neurological Surgery, University of California, San Francisco, San Francisco, CA, United States

**Keywords:** clinical trials, best practices, deep brain stimulation, neuropsychiatric diseases, major depressive disorder, neuroethics

## Abstract

Deep brain stimulation (DBS) is well suited to target disorders with network dysregulation, as is the case in many neuropsychiatric diseases. While DBS is a well-established therapy for Parkinson’s disease, essential tremor, dystonia, and medically refractory epilepsy, it is actively being studied in clinical trials for neuropsychiatric disorders including treatment-refractory major depressive disorder (MDD). Due to the nature of symptomology and participant characteristics, special care must be taken in the design and implementation of clinical trials testing DBS for neuropsychiatric disorders. In particular, these studies typically include multi-year relationships between participants and study staff with frequent interactions, high burden of study activities on participants, and disclosure by participants of sensitive information related to symptoms and disease state. Through our experience with six participants across more than 5 years of the Presidio clinical trial assessing personalized closed-loop DBS for treatment-refractory MDD, we have gathered experience and evidence to inform best practices for conducting these interaction-intensive clinical studies in a vulnerable population. Here, we present these Key Practices along with discussion, informed by multiple fundamental principles: The Belmont Report; emotional and physical safety for study participants and staff; and integrity and validity of scientific outcomes.

## Introduction

To ensure the protection and dignity of participants, staff of clinical trials involving human subjects must be guided by the foundational principles of Respect for Persons, Beneficence, and Justice presented in the Belmont Report (U.S. Department of Health and Human Services, 1979). These principles recognize the autonomy of individuals who have the right to make informed decisions about their participation in research, require researchers to maximize benefits and minimize harm to participants, and advocate for fairness in the distribution of the benefits and burdens of research. Specific to deep brain stimulation (DBS), there have been important publications about additional ethical considerations, including outlining the need for risk/benefit analyses, carefully considered inclusion and exclusion criteria, respect for participant autonomy and quality of life, and concerns with recording neural activity (Baker et al., 2023; Bell et al., 2009; Bell et al., 2016; Bell et al., 2011; Bell and Racine, 2013; Fins et al., 2011; Muñoz et al., 2020; Nuttin et al., 2014; Rabins et al., 2009; Acevedo et al., 2022; Synofzik and Schlaepfer, 2011; Park et al., 2017). Here, we provide an additional resource to support researchers involved in clinical trials testing DBS by presenting key considerations to foster productive professional relationships between participants and study staff, maximize benefit and minimize harm to DBS participants, and protect the integrity of trial results by conducting the trial using a scientifically rigorous, explicit protocol. We start by providing background on the current landscape of clinical trials testing DBS for neuropsychiatric indications, particularly the types of activities involved in many of these trials and the composition of study teams required for safely conducting these trials. We then discuss five Key Practices we believe are critical for the success of DBS trials in neuropsychiatric indications: (1) Setting expectations with study participants; (2) delineating scope of study staff responsibilities; (3) establishing and maintaining appropriate boundaries; (4) being mindful of dual-roles; and (5) involving the participant, their family, and caregivers.

## Background on clinical trials of DBS for neuropsychiatric indications

DBS involves the surgical implantation of electrodes in the brain and an implantable pulse generator to deliver therapeutic stimulation to targeted brain regions (Miocinovic et al., 2013; Lozano et al., 2019; Herrington et al., 2016). DBS is FDA-approved for Parkinson’s disease (PD) (Hacker et al., 2020; Fang and Tolleson, 2017) and has an FDA humanitarian device exemption (HDE) for the treatment of dystonia and Obsessive Compulsive Disorder (OCD) (U.S. Food and Drug Administration, 2009; U.S. Food and Drug Administration, 2003). It is being actively studied in clinical trials for multiple neuropsychiatric indications (Table 1). Individuals eligible for DBS trials have severe presentations of their disorder with high degrees of impairment and have typically received little to no benefit from standard-of-care treatments. Some may have developed distrust of the medical system or hopelessness for potential symptom relief.

**Table 1:** Clinical trials testing DBS for neuropsychiatric indications that are currently active (recruiting or not recruiting), based on search of clinicaltrials.gov in February 2025.

NCT number	Study status	Indication	Sponsor institution	Study design	Stimulation regions/conditions
NCT05245643	Recruiting	AN	Centre Hospitalier St Anne	Open-label single group	NAc
NCT01924598	Active, NR	AN	University of Oxford	Open-label single group	NAc
NCT06529380	Recruiting	ASD|SIB	The Hospital for Sick Children	Double-blind randomized crossover	Bilateral NAc vs. Sham
NCT05884619	Recruiting	AUD	Second Xiangya Hospital of Central South University	Open-label single group	Bilateral NAc and ALIC
NCT03660124	Active, NR	AUD	Sunnybrook Health Sciences Centre	Open-label single group	NAc
NCT06599099	Recruiting	BD	Baylor College of Medicine	Double-blind non-randomized parallel	Bilateral VC/VS-BNST vs. Sham
NCT05558358	Recruiting	MUD	University of Colorado, Denver	Double-blind randomized crossover	Bilateral NAc vs. Sham
NCT04281134	Active, NR	OCD	Baylor College of Medicine	Double-blind non-randomized parallel	VS DBS
NCT03457675	Active, NR	OCD	Baylor College of Medicine	Double-blind non-randomized parallel	VS
NCT04806516	Active, NR	OCD	Baylor College of Medicine	Double-blind non-randomized parallel	VS
NCT00640133	Active, NR	OCD	Butler Hospital	Double-blind randomized parallel	VC/VS vs. Sham
NCT03184454	Active, NR	OCD	Massachusetts General Hospital	Open-label single group	Dorsolateral PFC and ventral ALIC/VS
NCT05577598	Recruiting	OCD	Medical University of Vienna	Double-blind randomized crossover	ALIC vs. Sham
NCT02773082	Recruiting	OCD	Northwell Health	Open-label single group	Ventral ALIC
NCT05995951	Recruiting	OCD	Rabin Medical Center	Double-blind randomized crossover	Anteromedial STN vs. Sham
NCT04967560	Recruiting	OCD	Shanghai Mental Health Center	Double-blind randomized parallel	Bilateral NAc and ALIC vs. Sham
NCT04217408	Active, NR	OCD	Sunnybrook Health Sciences Centre	Double-blind randomized crossover	Open label bilateral VC/VS followed by VC/VS vs. Sham crossover
NCT06628752	Recruiting	OCD	Umeå University	Double-blind randomized parallel	BNST vs. Sham
NCT02377375	Recruiting	OCD	Universitaire Ziekenhuizen KU Leuven	Double-blind, randomized parallel	BNST
NCT02844049	Recruiting	OCD	University Hospital, Grenoble	Open-label randomized parallel	STN DBS with BMT vs. BMT
NCT06347978	Recruiting	OCD	University of California, San Francisco	Double-blind randomized crossover	Individualized site vs. Sham
NCT04958096	Recruiting	OCD	University of California, San Francisco	Open-label non-randomized parallel	ALIC, ALIC + OFC or ALIC + ACC
NCT05623306	Recruiting	OCD	University of Pennsylvania	Double-blind randomized crossover	Individualized site vs. Sham
NCT06542224	Recruiting	OCD	West China Hospital	Open-label single group	Bilateral DBS, region not specified
NCT04354077	Recruiting	OUD	Allegheny Singer Research Institute	Open-label single group	NAc
NCT05903495	Active, NR	OUD	West Virginia University	Double-blind randomized crossover	NAc and VC vs. Sham
NCT03950492	Active, NR	OUD	West Virginia University	Open-label single group	NAc and VC
NCT06705296	Recruiting	PTSD	Sunnybrook Health Sciences Centre	Double-blind non-randomized crossover	SGC: Open-label DBS, Double-blinded “on/off” DBS, Prolonged exposure therapy, Closed-loop DBS
NCT03416894	Active, NR	PTSD	Sunnybrook Health Sciences Centre	Open-label single group	SGC
NCT02091843	Recruiting	PTSD	VA Greater Los Angeles Healthcare System	Double-blind randomized parallel	Basolateral nucleus of the amygdala
NCT06423430	Recruiting	TRD	Abbott Medical Devices	Double-blind randomized parallel	Bilateral SCC white matter
NCT03437928	Recruiting	TRD	Baylor College of Medicine	Open-label single group	Individualized site
NCT00367003	Active, NR	TRD	Emory University	Open-label single group	Cg25
NCT01984710	Active, NR	TRD	Emory University	Open-label single group	Cg25
NCT04106466	Active, NR	TRD	Icahn School of Medicine at Mount Sinai	Open-label single group	SCC
NCT05773755	Recruiting	TRD	Icahn School of Medicine at Mount Sinai	Open-label single group	SCC
NCT06096207	Recruiting	TRD	Northwell Health	Double-blind randomized parallel	Superolateral MFB vs. Sham
NCT04530942	Active, NR	TRD	Ruijin Hospital	Double-blind randomized crossover	BNST-NAc vs. Sham
NCT06784388	Recruiting	TRD	Second Affiliated Hospital, School of Medicine, Zhejiang University	Open-label single group	Individualized site
NCT04009928	Recruiting	TRD	Sunnybrook Health Sciences Centre	Double-blind randomized crossover	MFB or SCC vs. Sham
NCT02046330	Active, NR	TRD	The University of Texas Health Science Center, Houston	Open-label single group	MFB
NCT03653858	Recruiting	TRD	University Hospital Freiburg	Double-blind randomized parallel	MFB vs. Sham
NCT04004169	Recruiting	TRD	University of California, San Francisco	Double-blind randomized crossover	Individualized site (biomarker controlled closed loop) vs. individualized site (fixed intermittent) vs. Sham
NCT03952962	Recruiting	TRD	University of Texas Southwestern Medical Center	Open-label randomized sequential	SCC
NCT06542094	Recruiting	TRD	West China Hospital	Open-label single group	Bilateral DBS, region not specified
NCT01798407	Active, NR	TRD|BD	Baylor College of Medicine	Double-blind non-randomized sequential	Bilateral LH
NCT02361554	Recruiting	TRS	Johns Hopkins University	Open-label single group	SNr
NCT05694000	Recruiting	TRS	Shanghai Mental Health Center	Double-blind randomized crossover	Bilateral ventral HPC vs. Sham
NCT06257056	Recruiting	TRS	University of Texas Southwestern Medical Center	Randomized sequential assignment	Individualized bilateral site; Open-label bilateral DBS, double blind discontinuation study

*Brain region: MFB, Medial forebrain bundle; BNST, Bed nucleus of stria terminalis; NAc, Nucleus accumbens; SGC, Subgenual cingulate; STN, Subthalamic nucleus; CAUD, Caudate nucleus; SNr, Substantia nigra pars reticulata; VC/VS, Ventral capsule/ventral striatum; Cg25, Subgenual cingulate; LH, Lateral habenula; ALIC, Anterior limb of the internal capsule; ACC, Anterior cingulate; PFC, Prefrontal cortex; SCC, Subcallosal cingulate; HPC, Hippocampus; OFC, Orbitofrontal cortex; BNST, Bed nucleus of the stria terminalis. Other: NR, Not recruiting; BMT, Best medical treatment; TRD, Treatment resistant depression; MUD, Methamphetamine use disorder; AUD, Alcohol use disorder; TRS, Treatment resistant schizophrenia; OUD, Opioid use disorder; BD, Bipolar depression; AN, Anorexia nervosa; NUD, Nicotine use disorder; SUD, Substance use disorder; PTSD, Posttraumatic stress disorder; ASD, Autism spectrum disorder; SIB, Self-injurious behavior.*

There are ethical and practical constraints to performing sham surgeries as a control condition, so randomized controlled trials of DBS often use a within-participant crossover design (Rabins et al., 2009), with each participant receiving both active and sham stimulation (AB/BA design). Stimulation parameters are sometimes optimized before the crossover begins, which introduces a substantial challenge for participants as they have experienced therapeutic stimulation but know that stimulation will be withheld during the crossover. Participants may experience heightened anxiety with upcoming start or switches of crossover arms and need to be reminded that stimulation will only be off temporarily. In addition, protocols should have explicit criteria for prematurely exiting a sham condition due to decompensation. The use of alternate study designs in which only one arm crosses from sham to active stimulation (AA/BA design) (Synofzik and Schlaepfer, 2011) has been proposed to avoid these concerns.

DBS requires ongoing monitoring and parameter adjustments to maximize benefits and ensure safety of participants. There are generally activities undertaken by the participant at home as well as study visits to monitor and change settings. For established indications such as PD, programming adjustments typically occur 3 to 11 times within the first six months following surgery (Ondo and Bronte-Stewart, 2005). Clinical trials assessing DBS for novel indications may require more frequent study visits and symptom reports to fully characterize therapeutic benefit or side-effects of therapy. Table 2 describes typical study activities and their frequency.

**Table 2:** Typical study activities for clinical trials testing DBS for neuropsychiatric indications.

Study purpose	Number of study visits	Activities
Recruitment and screening	1–10 virtual visits	Medical record review to determine eligibility
Consent	2 in-person visits	Teach-back consent over two separate visits; baseline questionnaires
Pre-surgery	3–8 in-person visits	Pre-surgical appointments with neurosurgery and anesthesia; (f)MRI scans
Surgery	2–3 in-person visits	2–3 day hospital admission for device implantation
Wound check	1 in-person visit	Surgical staples removed and wound check
Surgical recovery	4–8 virtual or in-person visits	1–2 months of surgical recovery; symptom assessment; education on device operation
Stimulation optimization	1–3 in-person visits/week	Stimulation safety testing and optimization
At-home data collection	1 virtual visit/week; daily participant uploads	Clinician scales to assess symptoms and side effects; participant upload data from device daily
Crossover phase	Weekly or every other week in-person visits	Clinician scales to assess symptoms and side effects; possibility of changing study arm
Long-term follow-up	Virtual or in-person visit every 6–8 weeks	Visits driven by participant desire and need for stimulation changes
Device explantation	2–3 in-person visits	If applicable, device explantation

## Study staff for clinical trials of DBS for neuropsychiatric indications

International psychiatric and neurosurgical societies have reached consensus that experienced multidisciplinary teams are mandatory for the ethical conduct of research on neuropsychiatric DBS or for therapeutic DBS offered through an HDE (Nuttin et al., 2014). Guidelines mandate teams with expertise from the following disciplines: stereotactic and functional neurosurgery, psychiatry, neurology, neuropsychology, and neuroethics. Based on our experience, the study team should also include well-trained clinical research coordinators (CRCs) (Buchanan et al., 2021) clinical psychologists, and if closed-loop stimulation is being employed (which involves analyzing neural activity to identify a symptom-correlated biomarker), individuals experienced in neurophysiology, neural signal processing, and decoding analyses. The study team must also have support for regulatory submissions to the FDA and IRB.

In our experience, CRCs play a critical role in DBS trials. CRCs are “on the front lines” and have the most day-to-day contact and communication with study participants (Davis et al., 2002). Therefore, CRCs are often the first to be aware of participant concerns. It is not a CRC’s job to de-escalate a participant in a crisis; rather, it is their job to recognize situations where participants need assistance and notify the proper study staff clinician(s) (Schatten et al., 2020) based on an established decision tree, as discussed below.

Having provided an overview of structure and design of clinical trials assessing DBS for neuropsychiatric indications, we next discuss the five Key Practices we believe are critical for successfully conducting these trials.

## Key practices

### Key practice 1: setting expectations with study participants

Clear and reiterated expectations are critical for successfully conducting DBS clinical trials. This includes (a) managing expectations the participant has about DBS and the clinical trial, and (b) establishing expectations the study team has of the participant.

DBS should not be thought of as a “cure-all” for the neuropsychiatric condition being investigated (Thomson and Carter, 2020). Participants who are eligible for neuropsychiatric DBS trials may have reached a point of desperation due to prior failed treatments and feel that DBS is their last and only hope for improvement (Thomson et al., 2021). However, DBS is only one aspect of a comprehensive treatment program (similar to traditional neuropsychiatric treatments), and participants may require extensive psychosocial rehabilitation (Nuttin et al., 2014; Rabins et al., 2009). Participants often contend with altered identity once stimulation alleviates disease symptoms which have been a core aspect of their lives for many years. Participants often have the desire to re-engage with education, employment, or social activities but are unable to make tangible steps toward these goals. Occupational or relational therapy and connecting with community services may be highly beneficial in these cases. We suggest creating a pamphlet that lists local, state, and federal agencies and support systems that can be distributed to both interested and enrolled participants.

There is always the possibility that DBS may be unsuccessful or only partly successful in mitigating disease symptoms for an individual. We have noticed that some participants drift in their mindset and think the study team has a ‘good’ setting for them and is ‘choosing’ to withhold that setting. While this may be true in the limited context of the sham condition during crossover periods, the study team may have been unsuccessful in identifying therapeutically beneficial stimulation or stimulation may have become less effective over time. During informed consent and following, direct conversations with the participant regarding all potential outcomes should be used to reorient and correct any therapeutic or trial misconceptions. Appropriate expectations may need to be reiterated several times, especially during a change in study condition or moving between symptomatic and asymptomatic states. A mnemonic has been deployed for DBS recipients for PD with success to aid in adjusting participants’ expectations to be more realistic (Okun and Foote, 2004). Lastly, participants must have accurate expectations that their contact with study staff will decrease and potentially cease as formal clinical trial activities are concluded.

It may be useful to create a ‘Research Engagement Agreement’, an IRB-approved document separate from the consent form. This agreement should be presented and discussed with the participant and their signature obtained; it can be referenced if corrective actions need to be taken. Topics which should be considered for inclusion in such an agreement include (a) Respectful engagement and non-discrimination; (b) Appropriate use of contact information; (c) Communication about study visit rescheduling; (d) Notification of any abrupt changes in symptoms or side effects believed to be related to stimulation; (e) Notification about changes in medical status or events which may impact the stimulation device (e.g., head impact). Finally, participants should know to contact 911 for any medical emergencies.

### Key practice 2: delineating scope of study staff responsibilities

Two key areas of study staff responsibility include managing suicide risk and long-term care considerations (Higgins et al., 2024; Dasgupta et al., 2024). Many neuropsychiatric clinical trials inquire about suicidality; the Columbia Suicide Severity Rating Scale is a commonly used instrument (Posner et al., 2011). A robust risk mitigation plan must be in place to respond to disclosure of suicidality from participants (Schatten et al., 2020). Assessing for suicide does not have a prospective iatrogenic effect (DeCou and Schumann, 2018), and should be conducted at every study interaction in clinical populations with a high propensity for suicide. If study visits occur remotely via telephone or video, participants should be asked where they are located in case it becomes necessary to send emergency services.

Each study should have an explicit decision tree to guide study staff following participant disclosure of suicidality. Participants determined to be at high-risk should be referred to study clinicians for immediate further evaluation. If the participant is in imminent danger of attempting suicide, ask the participant to call 911 or admit themselves to a hospital emergency department. Having the participant take this action themselves helps to preserve participant autonomy; however, if the participant is unwilling or unable to do so, study staff should contact emergency services directly to ensure participant safety. Clinicians have additional responsibilities based on the ethical principle of non-abandonment, which obligates them to follow care for the participant longitudinally or until transfer of care to a qualified clinician occurs (Nuttin et al., 2014).

Implanted DBS devices can remain functional for a decade or longer (Sette et al., 2019; Van Riesen et al., 2016). While the formal study activities for clinical trials typically do not last this long, a plan for long-term management of implanted DBS devices should be included in the trial design (Sankary et al., 2020). Although follow-up phases of 10 years or longer are being advised to better understand the long-term effects of DBS, it is unclear how entities can reliably financially support ongoing treatment maintenance over such a long period (Rabins et al., 2009). In some cases, participant care can be transferred to established clinics. However, due to either the types of devices implanted or the indication, some study participants may need to be followed indefinitely by the study team if therapy remains on. If such long-term monitoring is not feasible, devices may need to be explanted or deactivated to ensure long-term safety for participants.

### Key practice 3: establishing and maintaining appropriate boundaries

Many clinical trials testing DBS for neuropsychiatric diseases involve multi-year relationships between participants and study staff. Building rapport and trust with study participants is important but must be balanced with maintaining appropriate boundaries. The relationships between study staff and participants are fundamentally unbalanced, similar to the imbalances in provider/patient relationships (Afolabi, 2015; Baca, 2011). Study staff are in a position of power given their ability to ‘gate-keep’ access to therapy. Study staff may ask participants numerous questions about their prior life traumas, symptoms, activities of daily living, relationships, and other personal topics as a way to track disease symptoms, treatment efficacy, and side effects. However, disclosure of this type of information from study staff about themselves to participants is largely inappropriate.

We recommend having a conversation early on with participants about boundaries between study staff and participants. This helps to establish that study staff will ask many questions of the participant, but should not be asked reciprocal questions, and will decline to provide comparable information about themselves. To further support maintaining appropriate boundaries, all phone and text communication between CRCs and study participants should be done using a dedicated study phone number or device. Participants can be provided with contact information for the Principal Investigator and study Clinical Psychologist along with instructions regarding who to contact in case of emergency or other needs.

Study visits should ideally be conducted with two or more staff members present with the study participant in a designated professional space, which facilitates appropriate boundaries and the safety of visits. Dependent upon inclusion criteria, participants may have the potential for aggressive language or behavior, and staff safety should be prioritized. DBS side effects may also include agitation and hyperactivity (Seritan et al., 2021). Conducting a visit with two staff members also allows one person to remain with a participant in crisis while the other contacts emergency assistance, if required. If study visits must be conducted independently, ensure that someone else on the study team knows the time and location of the visit and have the in-person staff member check in with this colleague during and following the study visit.

### Key practice 4: being mindful of dual-roles

There are two forms of dual-roles involved in clinical trials for DBS. Individuals receiving treatment are both *participants* and *patients*, and the study staff includes those who are *researchers* and *clinicians*. The duality of these roles must be carefully monitored and care must be taken not to exceed the scope that is appropriate within a clinical trial.

People receiving DBS in clinical trials are by definition research *participants*. They give informed consent to research activities which follow an approved protocol. As such, they have the right to decline to perform study activities and can withdraw from the study at any time without penalty. If participants do decide to withdraw, special considerations may be needed to ensure long-term safety. This may involve explantation of the device system, turning off active stimulation, or establishing the individual with a clinic which can manage ongoing DBS therapy.

People receiving DBS in clinical trials are treated as *patients* in the context of device implantation (appointments with neurosurgery and anesthesia, hospital admission, pain management during the postoperative period, follow-up wound check). These participants receive medical care in the context of the clinical trial, but they should not receive other medical care from the study team. As such, we encourage verbiage such as ‘study visit’ rather than ‘clinic visit’ and ‘participant call’ vs. ‘telehealth appointment’ when conducting study activities. Of note, ensuring safety and directing participants to resources in the context of suicidality is within the scope of clinical trial activities and is mandated by the Belmont Report.

*Researchers* are responsible for contributing to the clinical trial protocol design, collecting and analyzing the data, and reporting results to clinical and research communities. These individuals may have other scientific interests which dovetail with DBS clinical trials. Some of these other research endeavors may be covered under separate IRB protocols which trial participants can decide if they want to participate in Thomson et al. (2021) and Morain et al. (2021). It should be made clear to trial participants which activities are related to the clinical trial (and therefore may directly benefit them if therapy is successful) versus other activities which do not have the potential for personal benefit. Declining to participate in ancillary activities should in no way negatively affect their participation in the clinical trial. Researchers must also ensure that research activities do not interfere with required clinical activities (e.g., those associated with DBS implantation).

*Clinicians* often switch roles between being a *researcher* and a *clinician* in DBS clinical trials (Mergenthaler et al., 2021). Clinicians provide medical care and assessments in the context of study activities, but they should not provide other medical care to study participants. Particular care must be taken if medications are prescribed in the context of the clinical trial. The trial-related purpose of these medications must be made abundantly clear, and any requests for renewal of other medications must be redirected to a non-study-related physician. Ongoing communication with a participant’s non-study-related medical providers may be required as other diagnoses or treatments may affect study activities, ongoing participant eligibility, or data interpretability.

### Key practice 5: involving the participant, their family, and caregivers

Clinical research is ideally a collaborative endeavor between study participants, their family, caregivers, and study staff. Inclusion in research development, trial processes, and social support not only helps participants comply with study activities, it also improves the safety profile of participation (Numans et al., 2019; Bird et al., 2020; Grady, 2022; Fins et al., 2017). We recommend that each study participant have a consented ‘study partner’ for the duration of the study. The study team can contact the study partner for additional information related to the participant’s symptoms or side effects and study partners serve as an important resource for support following surgery and in case of emergency (Thomson and Segrave, 2017; Thomson et al., 2023).

Because participants are central to DBS research (Acevedo et al., 2023), we have found it helpful to periodically offer continuing education and updates to participants about the utility of the data they provide, especially as repeated symptom reports over months can be burdensome and lead to participation fatigue. The principle of respect for persons requires informing research participants of study results if they are interested (Rabins et al., 2009). Participants often demonstrate interest in study procedures and are curious about how their experience maps onto the data and decisions of the research team (De Haan et al., 2015; Klein et al., 2016). Quarterly updates and end-of-year reviews that contain information about the quantity of data participants have provided, the team’s scientific output, and next steps can serve as an opportunity to thank participants for their hard work and share publicly available results with participants. However, the specifics of how much information is shared and when this occurs are study specific, and some study results should only be shared after full study completion. For example, unblinding an individual study participant’s crossover condition order may inadvertently unblind the conditions of other participants given small sample sizes and typically counterbalanced conditions across the sample within a study. Unblinding may also bias study staff and how they interact with future participants. With regard to blinding, many participants experience high anxiety leading up to the start of the crossover periods, and we have found that a blinded onset to a potential setting change can be beneficial to mitigate this anxiety and further aid in blinding staff involved in symptom ratings and clinician scales. Also note that while it is imperative for study staff to protect the privacy of research participants, participants are free to publicly disclose their participation in a clinical trial, such as via social media or in DBS support groups. Therefore, if premature release of information may be damaging to the integrity of the study results or the experience of other participants in the study, sharing results with participants may need to be delayed.

## Discussion

Clinical trials for DBS span early feasibility studies to pivotal trials. These trials require careful consideration due to the vulnerable population, implanted devices, sensitive nature of disclosures, and longitudinal nature of the treatment. Here, we provide five Key Practices for the successful completion of DBS trials in neuropsychiatric conditions. These Key Practices were developed from over half a decade’s worth of experience with participants in an intensive DBS clinical trial testing closed-loop DBS for MDD. The Key Practices were created with the goals of ethically *conducting* and *completing* clinical trials, but they do not ensure a positive outcome in terms of beneficial therapy. These Practices are not an exhaustive list of the ethical and practical considerations for successful DBS clinical trials, but we hope they support current work and foster continued discussion. As DBS technology and therapy continue to evolve, there is an ongoing need to critically evaluate how to conduct clinical trials responsibly to maximize benefit, minimize risk, and maintain scientific rigor.

## Data Availability

The original contributions presented in the study are included in the article/supplementary material, further inquiries can be directed to the corresponding author.
